# Risk factors for intensive care unit admission among COVID-19 patients in the West Bank, Palestine: a retrospective cohort study

**DOI:** 10.3389/fmed.2025.1634256

**Published:** 2025-09-11

**Authors:** Danyah Khalid, Hamzeh Al Zabadi, Jamal Qaddumi, Ibrahim Taha

**Affiliations:** ^1^Public Health Management, Faculty of Graduate Studies, An-Najah National University, Nablus, Palestine; ^2^Public Health Department, Faculty of Medicine and Health Sciences, An-Najah National University, Nablus, Palestine; ^3^Department of Nursing, Faculty of Medicine and Health Sciences, An-Najah National University, Nablus, Palestine; ^4^Optometry Department, Arab American University, Ramallah, Palestine

**Keywords:** COVID-19, ICU admission, risk factors, Palestine, retrospective cohort, obesity, Cox-regression

## Abstract

**Background:**

COVID-19 has significantly strained healthcare systems worldwide, particularly in resource-limited and conflict-affected regions such as Palestine. Understanding the risk factors for intensive care unit (ICU) admission is essential for effective triage and resource allocation.

**Objective:**

This study aimed to identify demographic, clinical, and laboratory predictors of ICU admission among hospitalized COVID-19 patients in governmental hospitals across the West Bank, Palestine.

**Methods:**

A retrospective cohort study was conducted across six designated COVID-19 treatment hospitals from November 2020 to February 2021. Medical records of 200 adult patients with PCR-confirmed COVID-19 were reviewed. Patients were grouped based on ICU versus general ward admission. Bivariate analyses, multivariate logistic regression, and Cox proportional hazards models were employed to identify independent predictors of ICU admission and to evaluate the time to ICU transfer.

**Results:**

Of the 200 patients, 117 (58.5%) were admitted to the ICU. Multivariate logistic regression identified obesity (OR = 66.7, *p* < 0.001), hospital site (*p* = 0.001), white blood cell count (OR = 1.25, *p* = 0.005), and blood urea nitrogen (OR = 0.96, *p* = 0.007) as significant positive predictors. Random blood sugar (OR = 0.99, *p* = 0.014) was inversely associated with ICU admission. Kaplan–Meier and Cox-regression analyses further revealed that obesity, low PaO₂, low BUN, low RBS, pregnancy, and pneumonia significantly shortened the time to ICU admission (all *p* < 0.05). Conversely, age, gender, comorbidities, and chief complaints were not independently associated with ICU admission.

**Conclusion:**

This is the first study in Palestine to comprehensively evaluate ICU admission risk factors among COVID-19 patients. Our findings can inform ICU triage protocols and help shape evidence-based health policies tailored to the Palestinian context and could establish data base for new similar future pandemics and/or resurge of COVID-19 for better emergency preparedness and intervention measures.

## Introduction

The novel coronavirus disease 2019 (COVID-19), caused by Severe Acute Respiratory Syndrome Coronavirus-2 (SARS-CoV-2), was first identified in December 2019 in Wuhan, China, and rapidly evolved into a global pandemic (1). Initially linked to zoonotic transmission from a seafood market in Wuhan, human-to-human transmission soon became evident, significantly accelerating its global spread (2). As of late 2024, the global impact of the COVID-19 pandemic has been substantial. According to the World Health Organization (WHO), over 776.8 million confirmed cases and more than 7 million confirmed deaths have been reported worldwide since the onset of the pandemic. However, the actual death toll is likely higher, with estimates suggesting that the true number of COVID-19-related deaths could exceed 20 million (3).

In Palestine, the first COVID-19 cases were reported in early March 2020. By late 2021, Palestine had confirmed over 465,000 COVID-19 cases, with approximately 4,900 fatalities, and more than 3,000 patients admitted to intensive care units (ICUs) in governmental hospitals (52). The rapid increase in critically ill COVID-19 patients presented significant challenges to the Palestinian healthcare system, highlighting its vulnerability due to limited ICU capacity and medical resources.

Identifying risk factors for ICU admission among COVID-19 patients is essential for developing targeted strategies to reduce morbidity and manage healthcare resources effectively. Prior international research indicated that various clinical and demographic factors significantly influenced the severity of COVID-19 infections. Key factors frequently associated with increased ICU admission risk include advanced age, male gender, obesity, smoking, diabetes, hypertension, cardiovascular and respiratory comorbidities, and specific laboratory abnormalities such as elevated inflammatory markers and abnormal coagulation profiles (2, 4, 5).

Numerous large-scale studies and meta-analyses have identified consistent predictors of ICU admission in COVID-19 patients. These include advanced age, male sex, obesity, cardiovascular disease, diabetes, elevated inflammatory markers (e.g., CRP, ferritin, LDH), lymphopenia, and abnormal coagulation parameters (6–8). A Prospective cohort study conducted by Cidade et al. (9) found that elevated triglycerides, procalcitonin, and CRP were among the top predictors of ICU admission. Similarly, other studies emphasized the prognostic value of D-dimer, neutrophilia, and LDH as strong laboratory indicators of disease severity (10).

Furthermore, clinical indicators such as hypoxia, elevated respiratory rate, and early need for oxygen supplementation have been consistently associated with ICU admission risk (11). These parameters inform triage algorithms in many healthcare systems. However, it is important to note that most of the available data derive from high-income countries with well-resourced healthcare infrastructures.

Recent studies from the MENA region have explored ICU predictors in COVID-19 patients. In Jordan, Mayyas et al. (12) found that ICU stay and mortality were associated with age, neutrophil-to-lymphocyte ratio, CRP, creatinine, and ventilation. An Egyptian study reported correlations with age, obesity, random blood sugar, inflammatory markers, and renal/liver dysfunction (13). Similarly, in Saudi Arabia, ICU admission was linked to age, diabetes, heart failure, CRP, and respiratory distress, while mortality was strongly predicted by oxygen desaturation, thrombocytopenia, and elevated troponin (14). While these studies offer valuable insights, most are limited by single-center design and lack multivariable adjustment. Palestine remains underrepresented, highlighting the need for context-specific data.

Furthermore, most existing ICU risk prediction models have been developed in high-income settings, like Palestine, with robust health infrastructure, limiting their applicability to health systems constrained by conflict, occupation, or chronic shortages in ICU capacity and diagnostics. Palestine, as a lower-middle-income setting under prolonged political and logistical strain, presents a unique case to explore how standard predictors perform in resource-limited environments. Insights from such settings are critical to closing global evidence gaps and informing more inclusive pandemic preparedness strategies worldwide.

Therefore, the aim of this study is to explicitly identify and evaluate the risk factors associated with ICU admission among COVID-19 patients hospitalized in governmental hospitals across the West Bank, Palestine. Specifically, the study seeks to assess demographic characteristics, clinical presentations, comorbidities, laboratory, and radiological findings, and treatment regimens as potential predictors for ICU admission. These findings are anticipated to inform clinical practice guidelines, support health policy decisions, and enhance preparedness to mitigate the impact of COVID-19 in Palestine.

## Methods

### Study design and setting

This study employed a retrospective cohort design to investigate risk factors associated with ICU admission among COVID-19 patients in the West Bank, Palestine. The research was conducted across six governmental hospitals designated as COVID-19 treatment centers, all equipped with intensive care units and operating under standardized clinical guidelines issued by the Palestinian Ministry of Health. These national guidelines defined ICU admission criteria based on oxygen saturation below 90% on room air, respiratory rate greater than 30 breaths per minute, hemodynamic instability, altered level of consciousness, or signs of multi-organ dysfunction. The study period extended from November 1, 2020, to February 1, 2021. A retrospective design was selected to utilize existing medical records, offering both time and resource efficiency while allowing for the analysis of multiple exposures and outcomes over a defined period.

### Study population and eligibility criteria

This retrospective cohort study included all adult patients (aged ≥18 years) with confirmed COVID-19 who were admitted to six governmental hospitals designated as COVID-19 treatment centers across the West Bank, Palestine, between November 1, 2020, and February 1, 2021. COVID-19 diagnosis was confirmed by reverse transcription polymerase chain reaction (RT-PCR). Patients were classified into two groups based on the level of care received: those admitted to intensive care units (ICUs) comprised the case group, while those treated in general COVID-19 wards served as the control group.

Inclusion criteria were: age 18 years or older; PCR-confirmed COVID-19 diagnosis; admission to either an ICU or a general ward during the study period; and availability of complete medical records within the Palestinian Health Information System (HIS), including demographic, clinical, laboratory, and treatment-related data. Patients were excluded if they had incomplete data on key clinical variables or outcomes, were managed solely in emergency departments without hospital admission, or were under 18 years of age.

### Sampling method and sample size

The study included all adult patients (≥18 years) with confirmed COVID-19 who were admitted to six designated governmental hospitals in the West Bank between November 1, 2020, and February 1, 2021. A total of 432 eligible patients were identified through hospital admission logs and medical records. No sampling technique was applied; rather, a complete enumeration (census) of all eligible patients during the study period was conducted based on the inclusion and exclusion criteria.

### Data collection tools and procedures

Data were extracted using a structured data extraction form developed by the research team. The form was based on findings from previous literature and was reviewed by two COVID-19 specialists working in the West Bank. After pilot testing and refinement, the final version of the tool captured five main domains: (1) demographic characteristics, including age, sex, smoking status, obesity, pregnancy, occupation, and hospital location; (2) medical history, such as comorbidities including diabetes, hypertension, and cardiovascular disease; (3) clinical features, including presenting symptoms and vital signs; (4) laboratory and radiological findings, such as complete blood count, renal function, inflammatory markers, oxygen saturation, and chest imaging results; and (5) treatment modalities, including antiviral use, antibiotic therapy, corticosteroids, oxygen therapy, and mechanical ventilation. For cases with incomplete records, additional demographic or clinical details were obtained by contacting surviving patients via telephone, provided contact information was available.

### Ethical and administrative considerations

Ethical approval for the study was granted by the Institutional Review Board (IRB) at An-Najah National University, as well as the scientific research committees of the participating institutions. Verbal informed consent was obtained from patients who were contacted by phone. All collected data were anonymized and securely stored, and were used solely for research purposes in accordance with ethical research guidelines.

### Statistical analysis

All statistical analyses were performed using IBM SPSS Statistics for Windows, Version 20.0 (IBM Corp., Armonk, NY, United States) and R (version 20.0) for *post hoc* modeling. Descriptive statistics were used to summarize the study population. Categorical variables were reported as frequencies and percentages, and continuous variables as means and standard deviations. Bivariate analyses were conducted to examine associations between independent variables and ICU admission. Chi-square tests were used for categorical variables, while independent-samples t-tests were applied to continuous variables. To identify independent predictors of ICU admission, multivariable logistic regression was performed.

### Variable selection criteria

Variables were included in the multivariable model based on (1) a *p*-value <0.20 in bivariate analysis to avoid excluding potentially meaningful predictors; (2) no evidence of multicollinearity or excessive variance inflation; and (3) established clinical or theoretical relevance as supported by existing literature and expert consensus. Adjusted odds ratios (AORs) and 95% confidence intervals (CIs) were reported, with *p*-values <0.05 considered statistically significant. Additionally, Cox proportional hazards regression was conducted to explore time-to-event outcomes (e.g., time to discharge or death), with results presented as hazard ratios (HRs) and 95% CIs. To address concerns about sparse data and inflated estimates—particularly for variables like obesity—a *post hoc* penalized logistic regression using Firth’s correction was performed in R. This method accounts for small-sample and quasi-complete separation bias. The Firth model showed strong overall significance (Likelihood Ratio Test = 104.28, *p* < 0.001; Wald test: *p* < 0.001) and produced more stable coefficient estimates.

Patients with missing data on key demographic, clinical, or outcome variables were excluded from the analysis. As a result, no imputation techniques were applied.

## Results

### Socio-demographic characteristics of participants

Out of the 200 hospitalized COVID-19 patients included in the study, 117 individuals (58.5%) required admission to the Intensive Care Unit (ICU), while 83 (41.5%) were treated in general wards. A significant association was found between ICU admission rates and several socio-demographic factors.

Regarding hospital of admission, approximately one-third of participants (31.5%) were treated at Bethlehem Hospital, followed by Dura Hospital and Tubas Hospital, each contributing 20% of cases. The remaining patients were distributed among Military Hospital (10.5%), Hugo Chavez Hospital (10%), and Red Crescent Hospital (5.5%). ICU admission rates varied significantly by hospital (*χ*^2^ = 20.195, *p* = 0.001), with Dura Hospital recording the highest ICU admission rate (79.5%), and Red Crescent the lowest (27.3%).

Occupational status was also a statistically significant factor (*p* = 0.010). The ICU admission rate was highest among unemployed individuals (73.8%), and lowest among healthcare workers (40%), suggesting that occupational exposure or socioeconomic context may influence disease severity or hospital trajectory.

Blood group also demonstrated a significant association with ICU admission (*p* = 0.036). Post-hoc analysis revealed that patients with blood group A+ had the highest ICU admission rate (81.3%), whereas group O+ patients had the lowest rate (43.6%).

Smoking status was another significant variable (*p* = 0.022). ICU admissions were more common among ex-smokers (70.1%) and current smokers (54.8%), compared to non-smokers (47.5%). This suggests a potential residual or cumulative risk effect from tobacco exposure.

Obesity emerged as one of the most powerful predictors of ICU admission. Among obese patients, 96.9% required ICU care, a markedly higher proportion than among non-obese patients (40.4%). Similarly, pregnancy was significantly associated with ICU admission, with 100% of pregnant patients in this cohort requiring intensive care (*p* < 0.001).

Other socio-demographic variables, including age group, sex, marital status, educational attainment, monthly income, and place of residence, did not show a statistically significant relationship with ICU admission (*p* > 0.05 for all). A full breakdown of the associations between demographic characteristics and ICU admission is provided in Table 1.

**Table 1:** Cross tabulation of demographic characteristics of COVID-19 patients’ participants and ICU admission.

Variable	Category		Endpoint		*χ^2^* (*p*-value)
		Total	Discharge	ICU admission	
		*n* (%)	*n* (%)	*n* (%)	
Hospital	Dura	44 (22.0)	9 (20.5)	35 (79.5)	**20.195(0.001)**
	Military	20 (10.0)	5 (25.0)	15 (75.0)	
	Red crescent	11 (5.5)	8 (72.7)	3 (27.3)	
	Hugo Chavez	21 (10.5)	7 (33.3)	14 (66.7)	
	Tubas	41 (20.5)	20 (48.8)	21 (51.2)	
	Bethlehem	63 (31.5)	34 (54.0)	29 (46.0)	
Age (year)	25–34	30 (15.0)	10 (33.3)	20 (66.7)	2.315(0.678)
	35–44	55 (27.5)	24 (43.6)	31 (56.4)	
	45–54	39 (19.5)	14 (35.9)	25 (64.1)	
	55–64	39 (19.5)	19 (48.7)	20 (51.3)	
	Above 65	37 (18.5)	16 (43.2)	21 (56.8)	
Gender	Male	80 (40.0)	33 (41.3)	47 (58.8)	0.003(0.953)
	Female	120 (60.0)	50 (41.7)	70 (58.3)	
Marital status	Single	78 (39.0)	39 (50.0)	39 (50.0)	4.696(0.195)
	Married	103 (51.5)	39 (37.9)	64 (62.1)	
	Divorced	8 (4.0)	2 (25.0)	6 (75.0)	
	Widowed	11 (5.5)	3 (27.3)	8 (72.7)	
Nature of work	Professional employee	73 (36.5)	37 (50.7)	36 (49.3)	**11.322(0.010)**
	Grafts man	46 (23.0)	18 (39.1)	28 (60.9)	
	Medical community	20 (10.0)	12 (60.0)	8 (40.0)	
	Not work	61 (30.5)	16 (26.2)	45 (73.8)	
Monthly income (shekel)	Below 1,500	71 (35.5)	28 (39.4)	43 (60.6)	3.970(0.137)
	1,500–3,000	67 (33.5)	34 (50.7)	33 (49.3)	
	3,000–4,500	62 (31.0)	21 (33.9)	41 (66.1)	
Education level	Illiterate	8 (4.0)	0 (0.0)	8 (100.0)	5.991(0.112)
	Primary school	63 (31.5)	28 (44.4)	35 (55.6)	
	High school	69 (34.5)	29 (42.0)	40 (58.0)	
	Diploma\B. A	60 (30.0)	26 (43.3)	34 (56.7)	
Blood group	A+	32 (16.0)	6 (18.8)	26 (81.3)	**15.034(0.036)**
	A-	20 (10.0)	8 (40.0)	12 (60.0)	
	AB+	17 (8.5)	5 (29.4)	12 (70.6)	
	AB-	21 (10.5)	9 (42.9)	12 (57.1)	
	O-	22 (11.0)	12 (54.5)	10 (45.5)	
	O+	39 (19.5)	22 (56.4)	17 (43.6)	
	B+	27 (13.5)	14 (51.9)	13 (48.1)	
	B-	22 (11.0)	7 (31.8)	15 (68.2)	
Place of residence	City	66 (33.0)	30 (45.5)	36 (54.5)	1.807(0.405)
	Village	71 (35.5)	25 (35.2)	46 (64.8)	
	Refugee camps	63 (31.5)	28 (44.4)	35 (55.6)	
Smoking status	Smoking	62 (31.0)	28 (45.2)	34 (54.8)	**7.650(0.022)**
	Ex-smokers	77 (38.5)	23 (29.9)	54 (70.1)	
	Non-smokers	61 (30.5)	32 (52.5)	29 (47.5)	
Obesity	Yes	64 (32.0)	2 (3.1)	62 (96.9)	**57.091(0.000)**
	No	136 (68.0)	81 (59.6)	55 (40.4)	
Pregnancy	Yes	21 (17.5)	0 (0.0)	21 (100.0)	**18.182(0.000)**
	No	99 (82.5)	50 (50.5)	49 (49.5)	

ICU, intensive care unit. Bold values indicate statistically significant results at *p* < 0.05.

### Medical history and ICU admission

Although the proportion of COVID-19 patients with a history of medical conditions was marginally higher among those admitted to the ICU compared to those treated in general wards, these differences were not statistically significant. Chi-square analysis revealed no significant association between ICU admission and the presence of comorbid conditions such as cardiac disease, chronic lung disease, diabetes, hypertension, chronic liver or renal disease, immunosuppression, dialysis status, blood disorders, neurological diseases, or endocrine disorders (all *p*-values > 0.05). These findings suggest that, in this cohort, pre-existing medical conditions alone were not sufficient predictors of ICU admission among hospitalized COVID-19 patients. Detailed data are presented in Table 2.

**Table 2:** Comparison of medical history of COVID-19 patients participants based on ICU admission.

Variable	Category	Total	Endpoint		χ^2^ (*p*-value)
			Discharge	ICU admission	
Cardiac disease	Yes	48 (24.0)	19 (39.6)	29 (60.4)	0.096(0.757)
	No	152 (76.0)	64 (42.1)	88 (57.9)	
Chronic lung disease	Yes	15 (7.5)	5 (33.3)	10 (66.7)	0.445(0.504)
	No	185 (92.5)	78 (42.2)	107 (57.8)	
Diabetes	Yes	70 (35.0)	27 (38.6)	43 (61.4)	0.380(0.537)
	No	130 (65.0%)	56 (43.1)	74 (56.9)	
Hypertension	Yes	62 (31.0%)	24 (38.7%)	38 (61.3%)	0.288(0.591)
	No	138 (69.0%)	59 (42.8%)	79 (57.2%)	
Chronic liver	Yes	5 (2.5%)	2 (40.0)	3 (60.0)	0.005(0.945)
	No	195 (97.5%)	81 (41.5)	114 (58.5)	
Chronic renal	Yes	3 (1.5%)	2 (66.7)	1 (33.3)	0.795(0.373)
	No	197 (98.5%)	81 (41.1)	116 (58.9)	
Immunosuppressed	Yes	7 (3.5%)	4 (57.1)	3 (42.9)	0.731(0.393)
	No	193 (96.5%)	79 (40.9)	114 (59.1)	
On dialysis	Yes	3 (1.5%)	2 (66.7)	1 (33.3)	0.795(0.373)
	No	197 (98.5%)	81 (41.1)	116 (58.9)	
Blood diseases	Yes	10 (5.0%)	3 (30.0)	7 (70.0)	0.573(0.449)
	No	190 (95.0%)	80 (42.1)	110 (57.9)	
Nervous diseases	Yes	3 (1.5%)	2 (66.7)	1 (33.3)	0.795(0.373)
	No	197 (98.5%)	81 (41.1)	116 (58.9)	
Endocrine diseases	Yes	3 (1.5%)	1 (33.3)	2 (66.7)	0.084(0.772)
	No	197 (98.5%)	82 (41.6)	115 (58.4)	

ICU, intensive care unit.

### Chief complaints and ICU admission

The association between presenting symptoms and ICU admission among COVID-19 patients was assessed using chi-square analysis. The results revealed that none of the commonly reported symptoms—including fever, cough, shortness of breath, chest pain, fatigue, malaise, diarrhea, headache, or sore throat—had a statistically significant relationship with ICU admission (all *p*-values > 0.05). However, vomiting was marginally associated with ICU admission, with a p-value of 0.053, suggesting a potential trend worth exploring further. Patients who presented with vomiting had a higher likelihood of ICU admission compared to those without this symptom. A detailed breakdown of symptoms and their association with ICU admission is provided in Table 3.

**Table 3:** Comparison of COVID-19 patients’ chief complains based on ICU admission versus discharge.

Chief complains		Total	Outcome		χ^2^ (*p*-value)
			Discharge	ICU admission	
Fever	Yes	170 (85.0%)	13 (43.3)	17 (56.7)	0.049(0.825)
	No	30 (15.0%)	70 (41.2)	100 (58.8)	
Joint pain	Yes	160 (80.0%)	17 (42.5)	23 (57.5)	0.021(0.886)
	No	40 (20.0%)	66 (41.2)	94 (58.8)	
Muscle pain	Yes	155 (77.5%)	19 (42.2)	26 (57.8)	0.012(0.911)
	No	45 (22.5%)	64 (41.3)	91 (58.7)	
Cough	Yes	140 (70.0%)	23 (38.3)	37 (61.7)	0.354(0.552)
	No	60 (30.0%)	60 (42.9)	80 (57.1)	
Confusion irritability	Yes	65 (32.5%)	56 (44.8)	69 (55.2)	1.571(0.456)
	No	125 (62.5%)	23 (35.4)	42 (64.6)	
	NA	10 (5.0%)	4 (40.0)	6 (60.0)	
Chills rigors	Yes	75 (37.5%)	41 (45.6)	49 (54.4)	2.308(0.315)
	No	90 (45.0%)	26 (34.7)	49 (65.3)	
	NA	35 (17.5%)	16 (45.7)	19 (54.3)	
Malaise	Yes	90 (45.0%)	38 (44.7)	47 (55.3)	0.938(0.626)
	No	85 (42.5%)	34 (37.8)	56 (62.2)	
	NA	25 (12.5%)	11 (44.0)	14 (56.0)	
Sore throat	Yes	125 (62.5%)	27 (41.5)	38 (58.5)	0.010(0.995)
	No	65 (32.5%)	52 (41.6)	73 (58.4)	
	NA	10 (5.0%)	4 (40.0)	6 (60.0)	
Diarrhea	Yes	65 (32.5%)	56 (46.7)	64 (53.3)	3.596(0.166)
	No	120 (60.0%)	21 (32.3)	44 (67.7)	
	NA	15 (7.5%)	6 (40.0)	9 (60.0)	
Shortness of breath	Yes	80 (40.0%)	54 (45.0)	66 (55.0)	1.514(0.219)
	No	120 (60.0%)	29 (36.3)	51 (63.7)	
Nausea	Yes	75 (37.5%)	56 (46.7)	64 (53.3)	3.714(0.156)
	No	120 (60.0%)	26 (34.7)	49 (65.3)	
	NA	5 (2.5%)	1 (20.0)	4 (80.0)	
Runny nose	Yes	65 (32.5%)	61 (45.2)	74 (54.8)	2.324(0.127)
	No	135 (67.5%)	22 (33.8)	43 (66.2)	
Vomiting	Yes	60 (30.0%)	62 (47.7)	68 (52.3)	**5.866(0.053)**
	No	130 (65.0%)	18 (30.0)	42 (70.0)	
	NA	10 (5.0%)	3 (30.0)	7 (70.0)	
Headache	Yes	130 (65.0%)	30 (42.9)	40 (57.1)	0.082(0.775)
	No	70 (35.0%)	53 (40.8)	77 (59.2)	
Conjunctivitis	Yes	40 (20.0%)	54 (45.0)	66 (55.0)	4.037(0.133)
	No	120 (60.0%)	11 (27.5)	29 (72.5)	
	NA	40 (20.0%)	18 (45.0)	22 (55.0)	
Fatigue	Yes	150 (75.0%)	20 (40.0)	30 (60.0)	0.062(0.804)
	No	50 (25.0%)	63 (42.0)	87 (58.0)	
Abdominal pain	Yes	80 (40.0%)	42 (46.7)	48 (53.3)	2.423(0.298)
	No	90 (45.0%)	28 (35.0)	52 (65.0)	
	NA	30 (15.0%)	13 (43.3)	17 (56.7)	
Loss of taste smell	Yes	140 (70.0%)	24 (40.0)	36 (60.0)	0.079(0.778)
	No	60 (30.0%)	59 (42.1)	81 (57.9)	
Chest pain	Yes	135 (67.5%)	26 (40.0)	39 (60.0)	0.089(0.765)
	No	65 (32.5%)	57 (42.2)	78 (57.8)	

ICU, intensive care unit. Bold values indicate statistically significant results at *p* < 0.05.

### Hemodynamics and laboratory results in relation to ICU admission

To explore the relationship between physiological and laboratory parameters and ICU admission, independent sample *t*-tests were conducted. The analysis revealed statistically significant differences between ICU-admitted and ward-managed COVID-19 patients in several key indicators:

- Oxygen saturation (SpO₂) was significantly lower among ICU patients (*p* = 0.040),
- Partial pressure of arterial oxygen (PaO₂) was also significantly reduced in ICU cases (*p* = 0.010),
- Blood urea nitrogen (BUN) levels showed a notable difference (*p* = 0.017),
- White blood cell (WBC) count was higher in ICU patients (*p* = 0.032),
- Random blood sugar (RBS) levels were significantly lower in ICU-admitted patients (*p* = 0.002).

These findings suggest that respiratory and metabolic deterioration were closely associated with the need for intensive care.

In addition, pneumonia was significantly more common among ICU patients, with 59.0% of pneumonia cases requiring ICU admission compared to only 41.0% in non-ICU patients (*χ*^2^ = 9.082, *p* = 0.003). This highlights pneumonia as a strong clinical marker for critical care needs in COVID-19 cases. Further details are presented in Table 4.

**Table 4:** Comparison of hemodynamics and lab result between COVID-19 patients admitted to ICU and discharge.

Variable	ICU admission	*N*	Mean	Std. D	*t*	*P*-value
Highest temp.	No	83	38.57	0.97	0.741	0.460
	Yes	117	38.47	0.83		
O2 saturation	No	83	92.14	3.29	**−2.069**	**0.040**
	Yes	117	93.14	3.41		
Temperature	No	83	37.93	0.79	−1.675	0.096
	Yes	117	38.12	0.80		
Heart Rate	No	83	93.40	17.95	0.755	0.451
	Yes	117	91.50	16.77		
Systolic BP	No	83	122.71	19.90	−1.518	0.131
	Yes	117	127.08	20.25		
Diastolic BP	No	83	68.34	14.96	−1.610	0.109
	Yes	117	71.85	15.58		
Resp. Rate	No	83	21.66	4.20	0.332	0.740
	Yes	117	21.46	4.24		
PaO2	No	83	105.22	21.13	**2.592**	**0.010**
	Yes	117	97.63	19.39		
PaCo2	No	83	40.25	5.40	−0.802	0.424
	Yes	117	40.86	5.17		
PH	No	83	7.39	0.10	0.757	0.450
	Yes	117	7.38	0.09		
HCO3	No	83	24.02	1.96	−0.393	0.695
	Yes	117	24.15	2.67		
ALT	No	83	46.68	19.52	1.725	0.086
	Yes	117	41.89	19.05		
AST	No	83	36.23	16.50	−1.587	0.114
	Yes	117	39.85	14.96		
BUN	No	83	33.04	18.23	**2.422**	**0.017**
	Yes	117	26.87	17.08		
CRE	No	83	1.46	0.55	1.684	0.094
	Yes	117	1.32	0.62		
D dimer	No	83	576.18	191.45	−0.883	0.379
	Yes	117	706.94	1585.43		
PTT	No	83	39.02	14.98	−1.239	0.217
	Yes	117	41.89	17.65		
PT	No	83	29.30	10.66	**1.808**	**0.072**
	Yes	117	26.54	10.65		
INR	No	83	2.09	0.81	0.978	0.330
	Yes	117	1.97	0.91		
Hb	No	83	13.19	1.81	0.844	0.400
	Yes	117	12.95	2.08		
Lymphocyte	No	83	1.42	0.54	−0.738	0.462
	Yes	117	1.58	2.30		
WBC	No	83	13.83	3.48	**−2.156**	**0.032**
	Yes	117	14.89	3.38		
Platelet	No	83	312.25	85.36	−0.210	0.834
	Yes	117	314.80	82.91		
Neutrophil	No	83	6.81	2.89	−1.419	0.158
	Yes	117	7.82	6.87		
HbA1c	No	83	1.80	0.41	0.579	0.564
	Yes	117	1.76	0.43		
RBS	No	83	214.25	90.68	**3.088**	**0.002**
	Yes	117	178.16	66.24		
LDH	No	83	267.53	72.32	−1.520	0.130
	Yes	117	282.65	83.61		
Ferritin	No	83	676.04	209.12	0.741	0.460
	Yes	117	628.91	233.43		

		Total	Discharge	ICU admission	*χ*^2^ (*p*-value)
Result_x-ray	Bilateral chest patchy shadow	90 (47.4)	32 (41.0)	58 (51.8)	2.135(0.144)
	Infiltration of lungs	100 (52.6)	46 (59.0)	54 (48.2)	
Result_CT	Presence of endpoint	4 (44.4)	0 (0.0)	4 (80.0)	**6.975(0.073)**
	Non-presence of endpoint	2 (22.2)	1 (25.0)	1 (20.0)	
	Pulmonary tissue swelling	2 (22.2)	2 (50.0)	0 (0.0)	
	Others	1 (11.1)	1 (25.0)	0 (0.0)	
Pneumonia	No	100 (50.0)	52 (62.7)	48 (41.0)	**9.082(0.003)**
	Yes	100 (50.0)	31 (37.3)	69 (59.0)	
Troponin1	Negative	36 (18.0)	72 (86.7)	92 (78.6)	2.166(0.141)
	Positive	164 (82.0)	11 (13.3)	25 (21.4)	
CRP	Positive	200 (100)	83 (100.0)	117 (100.0)	NA

ICU, intensive care unit; O₂ saturation, oxygen saturation; PaO₂, partial pressure of arterial oxygen; PaCO₂, partial pressure of arterial carbon dioxide; BP, blood pressure; ALT, alanine aminotransferase; AST, aspartate aminotransferase; BUN, blood urea nitrogen; CRE, creatinine; PT, prothrombin time; PTT, partial thromboplastin time; INR, international normalized ratio; Hb, hemoglobin; WBC, white blood cells; RBS, random blood sugar; LDH, lactate dehydrogenase; CRP, C-reactive protein; CT, computed tomography; NA, not applicable. Bold values indicate statistically significant results at *p* < 0.05.

### Medication use and ICU admission

The association between commonly administered medications and ICU admission was evaluated using chi-square analysis. The results revealed that there was a statistically significant relationship between the use of vancomycin, meropenem, and Actemra (tocilizumab) and the likelihood of ICU admission among hospitalized COVID-19 patients (*p* = 0.035, 0.008, and <0.001, respectively). Specifically, patients who received vancomycin or meropenem had higher ICU admission rates (67.5 and 70.1%) compared to those who did not receive these drugs (52.5 and 51.2%, respectively). In contrast, patients treated with Actemra were less likely to be admitted to the ICU, with only 7.7% of those who received it requiring intensive care compared to 62.0% among those who did not—indicating a potentially protective effect. Although there were small differences in antibiotic usage between ICU and non-ICU groups, no statistically significant relationship was found between the use of other antibiotics (e.g., ceftriaxone, levofloxacin, azithromycin) and ICU admission (all *p*-values > 0.05). Detailed comparisons by medication type are presented in Table 5.

**Table 5:** Comparison of drugs used by COVID-19 patients’ participants based on outcome (discharge, ICU admission).

Variable	Category	Outcome		*X*^2^(*p*-value)
		Discharge	ICU admission	
Vancomycin	No	57 (47.5%)	63 (52.5%)	**4.449(0.035)**
	Yes	26 (32.5%)	54 (67.5%)	
Meropenem	No	60 (48.8%)	63 (51.2%)	**6.975(0.008)**
	Yes	23 (29.9%)	54 (70.1%)	
Ciprofloxacin	No	82 (41.6%)	115 (58.4%)	0.084(0.772)
	Yes	1 (33.3%)	2 (66.7%)	
Ceftriaxone	No	52 (46.4%)	60 (53.6%)	2.547(0.111)
	Yes	31 (35.2%)	57 (64.8%)	
Tazocin	No	73 (42.0%)	101 (58.0%)	0.034(0.853)
	Yes	10 (40.0%)	15 (60.0%)	
Levofloxacin	No	14 (45.2%)	17 (54.8%)	0.203(0.653)
	Yes	69 (40.8%)	100 (59.2%)	
Azithromycin	No	3 (21.4%)	11 (78.6%)	2.498(0.114)
	Yes	80 (43.0%)	106 (57.0%)	
hydrocortisone	No	37 (43.0%)	49 (57.0%)	0.144(0.704)
	Yes	46 (40.4%)	68 (59.6%)	
Cough syrup	No	23 (39.7%)	35 (60.3%)	0.115(0.735)
	Yes	60 (42.3%)	82 (57.7%)	
Vitamin C	No	25 (41.0%)	36 (59.0%)	0.010(0.922)
	Yes	58 (41.7%)	81 (58.3%)	
Vitamin D	No	15 (39.5%)	23 (60.5%)	0.079(0.778)
	Yes	68 (42.0%)	94 (58.0%)	
Zinc	No	13 (32.5%)	27 (67.5%)	1.668(0.196)
	Yes	70 (43.8%)	90 (56.3%)	
Acetylcysteine	No	59 (39.9%)	89 (60.1%)	0.627(0.428)
	Yes	24 (46.2%)	28 (53.8%)	
Enoxaparin	No	4 (30.8%)	9 (69.2%)	0.659(0.417)
	Yes	79 (42.2%)	108 (57.8%)	
PI	No	2 (50.0%)	2 (50.0%)	0.121(0.727)
	Yes	81 (41.3%)	115 (58.7%)	
Corticosteroids Neb	No	3 (42.9%)	4 (57.1%)	0.006(0.941)
	Yes	80 (41.5%)	113 (58.5%)	
Paracetamol	No	26 (41.9%)	36 (58.1%)	0.007(0.933)
	Yes	57 (41.3%)	81 (58.7%)	
Colchicine	No	76 (41.8%)	106 (58.2%)	0.056(0.814)
	Yes	7 (38.9%)	11 (61.1%)	
Actemra	No	71 (38.0%)	116 (62.0%)	**14.784(0.000)**
	Yes	12 (92.3%)	1(7.7%)	

ICU, intensive care unit; X^2^, Chi-square; PI, proton inhibitor; Neb, nebulized; Actemra, tocilizumab. Bold values indicate statistically significant results at *p* < 0.05.

### COVID-19 patients’ predictors of ICU admission

To identify the independent predictors of ICU admission among COVID-19 patients, variables were entered into the multivariate logistic regression model analysis based on the following criteria: firstly, they should be statistically significant or *p*-value of <0.20 in bivariate analyses and should not affect the total variance of the model. Finally, through literature and expert discussion as being theoretical and clinical relevance variable. The aim was to control for confounding factors and determine the adjusted effects of each variable.

The goodness-of-fit using the Hosmer–Lemeshow test indicated that the model adequately fits the data, *χ*^2^(8) = 4.747, *p* = 0.784.

The analysis revealed that hospital type (*p* = 0.001), obesity (*p* < 0.001), blood urea nitrogen (BUN; *p* = 0.007), white blood cell count (WBC; *p* = 0.005), and random blood sugar (RBS; *p* = 0.014) were all statistically significant predictors of ICU admission.

Patients admitted to Dura Hospital were 5.70 times more likely to be admitted to the ICU compared to patients from other hospitals (OR = 5.70, 95% CI: 2.104–15.451). Obese patients were remarkably more likely to require ICU care, with an odds ratio of 66.736 (95% CI: 12.723–350.066), highlighting obesity as a major risk factor. Furthermore, each unit increase in WBC was associated with a 1.245-fold increase in the odds of ICU admission (95% CI: 1.070–1.448).

Conversely, higher BUN and RBS levels were associated with a reduced likelihood of ICU admission. Specifically, the odds of ICU admission decreased by 3.9% for every unit increase in BUN (OR = 0.961, 95% CI: 0.933–0.989), and by 0.8% for every unit increase in RBS (OR = 0.992, 95% CI: 0.986–0.998).

Although vomiting showed a higher odds ratio (OR = 1.508, 95% CI: 0.580–3.922), it did not reach statistical significance (*p* = 0.400), and therefore could not be confirmed as an independent predictor of ICU admission (see Table 6).

**Table 6:** Predictors of ICU admission among COVID-19 patients.

Variable	*B*	S. E.	Wald	df	Sig.	AOR	95% CI for AOR	
							Lower	Upper
Hospital			19.602	5	**0.001**			
Dura	1.741	0.509	11.713	1	0.001	5.702	2.104	15.451
Military	0.393	0.740	0.281	1	0.596	1.481	0.347	6.320
Red crescent	−2.829	1.351	4.385	1	0.036	0.059	0.004	0.834
Hugo Chavez	0.000	0.739	0.000	1	1.000	1.000	0.235	4.257
Tubas	−0.048	0.503	0.009	1	0.925	0.953	0.355	2.557
Nature of work	−0.187	0.203	0.850	1	0.357	0.830	0.558	1.234
Blood group	−0.120	0.088	1.858	1	0.173	0.887	0.746	1.054
Smoking status	−0.050	0.235	0.046	1	0.831	0.951	0.600	1.506
Obesity	4.641	1.047	19.668	1	**0.000**	66.736	12.723	350.066
Vomiting	0.411	0.488	0.709	1	0.400	1.508	0.580	3.922
O2 saturation	0.125	0.071	3.105	1	0.078	1.133	0.986	1.302
PaO2	−0.023	0.012	3.592	1	0.058	0.977	0.954	1.001
BUN	−0.040	0.015	7.352	1	**0.007**	0.961	0.933	0.989
WBC	0.219	0.077	8.014	1	0.**005**	1.245	1.070	1.448
RBS	−0.008	0.003	6.001	1	**0.014**	0.992	0.986	0.998
Pneumonia	0.306	0.498	0.378	1	0.539	1.359	0.512	3.608
Constant	−7.638	6.503	1.379	1	0.240	0.000		

ICU, intensive care unit; B, regression coefficient; S. E., standard error; df, degrees of freedom; Sig., significance (*p*-value); AOR, adjusted odds ratio; CI, confidence interval; O₂ saturation, oxygen saturation; PaO₂, partial pressure of oxygen; BUN, blood urea nitrogen; WBC, white blood cell count; RBS, random blood sugar. Bold values indicate statistically significant results at *p* < 0.05.

It is important to note that other variables previously significant in the bivariate analysis lost their predictive power when adjusted for in the multivariate model. This suggests that their initial associations may have been confounded by other factors.

Firth correction analysis using r program reflect that the model is statistically significant overall (LRT = 104.28, *p* < 0.001, Wald test: *p* < 0.001), and the Firth correction worked in stabilizing estimates like obesity and found a strong association (OR = exp. [3.697], CI = [11 to 228]) of obesity with the outcome, but still reflect sparse data as very few obese patients admitted to ICU. Also, the sensitivity analysis support the robustness of several associations and highlight the need to interpret obesity’s impact carefully. The revised model continued to show statistically significant predictors of ICU admission.

### Kaplan–Meier survival analysis and Cox regression of ICU admission predictors

Kaplan–Meier survival curves and log-rank tests were used to compare differences in time to ICU admission across several patient characteristics, including obesity, blood group, employment status, smoking, pregnancy, pneumonia, and selected laboratory indicators (PaO₂, BUN, and RBS). Subsequently, univariate Cox proportional hazards regression was employed to examine the associations between these covariates and time to ICU admission.

Obesity was significantly associated with earlier ICU admission. Obese patients had a mean time to ICU admission of 2.3 days, significantly shorter than the 9.2 days observed in non-obese patients (*p* < 0.001). Similarly, patients who were unemployed were admitted to ICU more quickly (mean = 5.3 days) compared to those working in the medical field (mean = 9.2 days; *p* = 0.026).

Patients with blood group A + had a significantly shorter time to ICU admission (mean = 3.9 days) than those with blood group O+ (mean = 8.2 days; *p* = 0.007). Smokers also tended to be admitted earlier (mean = 7.3 days) than non-smokers (mean = 8.3 days), though the difference approached but did not reach statistical significance (*p* = 0.056).

Pregnant patients demonstrated a significantly earlier ICU admission compared to non-pregnant counterparts (mean = 2.0 vs. 7.6 days; *p* < 0.001). Likewise, patients diagnosed with pneumonia were admitted earlier (mean = 5.7 days) than those without pneumonia (mean = 8.3 days; *p* = 0.001).

Univariate Cox regression analysis confirmed that lower PaO₂ (*p* = 0.017), lower BUN (*p* = 0.041), and lower RBS levels (*p* = 0.010) were significantly associated with earlier ICU admission among COVID-19 patients (see Table 7).

**Table 7:** Univariate Cox-regression analysis of factors contributing to time in ward till admitted to ICU.

Variable	Category	Mean		95% CI		Log rank
		Estimate	Std. error	Lower bound	Upper bound	χ^2^ (*p*-value)
Obesity	No	9.201	0.501	8.218	10.183	**61.903(0.000)**
	Yes	2.336	0.217	1.910	2.762	
	Overall	7.039	0.417	6.222	7.857	
Work	Not work	5.335	0.670	4.022	6.648	**0.274(0.026)**
	Professional employee	8.055	0.709	6.665	9.444	
	Graftsman	6.696	0.868	4.994	8.398	
	Medical community	9.350	1.277	6.847	11.853	
	Overall	7.039	0.417	6.222	7.857	
Blood group	A+	3.958	0.779	2.432	5.485	**19.452(0.007)**
	A-	6.950	1.293	4.415	9.485	
	AB+	5.529	1.339	2.905	8.154	
	AB-	7.286	1.274	4.788	9.784	
	O-	8.000	1.175	5.697	10.303	
	O+	8.282	0.864	6.588	9.976	
	B+	8.460	1.116	6.273	10.647	
	B-	5.818	1.200	3.466	8.171	
	Overall	7.039	0.417	6.222	7.857	
Smoking	Current smoking	7.339	0.771	5.827	8.851	**5.779(0.056)**
	Ex-smokers	5.735	0.621	4.519	6.952	
	Non-smokers	8.386	0.759	6.899	9.874	
	Overall	7.039	0.417	6.222	7.857	
Pregnancy	No	7.619	0.446	6.745	8.493	**17.497(0.000)**
	Yes	2.095	0.206	1.692	2.499	
	Overall	7.039	0.417	6.222	7.857	
Pneumonia	No	8.369	0.590	7.213	9.524	**11.051 (0.001)**
	Yes	5.710	0.560	4.613	6.807	
	Overall	7.039	0.417	6.222	7.857	

	B	Wald	df	Sig.	Exp(B)	CI for OR
PaO2	−0.011	5.721	1	**0.017**	0.989	0.980–0.998
BUN	−0.011	4.164	1	**0.041**	0.989	0.979–1.000
RBS	−0.003	6.555	1	**0.010**	0.997	0.994–0.999

CI, confidence interval; Std. error, standard error; *χ*^2^ (*p*-value), Chi-square test with *p*-value; B, regression co-efficient; Wald, Wald Test statistic; df, degrees of freedom; Sig., significance (*p*-value); Exp(B), Hazard ratio; PaO₂, partial pressure of arterial oxygen; BUN, blood urea nitrogen; RBS, random blood sugar. Bold values indicate statistically significant results at *p* < 0.05.

Moreover, Table 8 summarizes the multivariate (adjusted) Cox proportional hazards regression for predictors/factors of COVID-19 patient’s ICU admission.

**Table 8:** Multivariate Cox regression analysis of factors contributing to time in ward till admitted to ICU.

Variable	*B*	Wald	Sig.	OR	95.0% CI for OR	
					Lower	Upper
Work	0.036	0.116	0.733	1.036	0.843	1.274
Blood group	−0.037	0.784	0.376	0.964	0.888	1.046
Smoking	−0.036	0.082	0.774	0.964	0.752	1.236
Obesity	1.143	27.574	**0.000**	3.135	2.047	4.803
Pregnancy	−0.293	0.962	0.327	0.746	0.415	1.340
Pneumonia	0.330	2.719	0.099	1.391	0.940	2.060
PaO2	−0.009	3.244	0.072	0.991	0.981	1.001
BUN	−0.010	2.741	0.098	0.990	0.979	1.002
RBS	−0.002	2.989	0.084	0.998	0.995	1.000

OR, odds ratio; B, regression coefficient; Wald, Wald Test statistic; Sig., significance (*p*-value); Exp(B), Hazard ratio; CI, confidence interval; PaO₂, partial pressure of arterial oxygen; BUN, blood urea nitrogen; RBS, random blood sugar. Bold values indicate statistically significant results at *p* < 0.05.

The calculated Variance Inflation Factors (VIFs) for independent variables were between 1.061 and 1.491, which were below 10, thus confirming the absence of multicollinearity.

After adjusting for the demographic as control variable, and predictors/factors of interest, obesity (HR (95% CI): 3.135 (2.047–4.803), *p* < 0.001) was found to be significant predictor for ICU admission among COVID-19 patients in ICU. For obesity, the HR of 3.135 indicates that the expected hazard (admission to ICU) of COVID-19 patients is 3.135 times than non-obese COVID-19 patients.

## Discussion

The coronavirus disease 2019 (COVID-19), caused by the novel SARS-CoV-2 virus, has strained healthcare systems globally, with particularly severe consequences in resource-limited and conflict-affected regions. Identifying patients at high risk for severe outcomes, such as intensive care unit (ICU) admission, is essential for optimizing resource allocation and guiding clinical decision-making (2, 5). While global studies have established several risk factors—including advanced age, obesity, comorbid conditions, and elevated inflammatory markers—there is a significant gap in context-specific data from low-income or politically unstable regions such as Palestine (15, 16). This study aims to fill that gap by investigating the predictors of ICU admission among hospitalized COVID-19 patients across multiple governmental hospitals in the West Bank, thereby providing localized insights to strengthen clinical triage strategies and public health policy.

In the current study, the nature of the patient’s occupation was found to be significantly associated with ICU admission among COVID-19 patients, a finding consistent with that of (17). Specifically, ICU admission was more common among unemployed individuals. This may be attributed to the economic impact of the pandemic, during which many people lost their jobs due to widespread lockdowns and the closure of businesses, factories, and public events. During this period, quarantine measures were enforced, and social activities such as weddings and family gatherings were halted. Only healthcare workers maintained consistent exposure due to their essential roles. While our study showed higher ICU admissions among the unemployed, prior research has reported greater ICU admissions among healthcare professionals (18). This discrepancy may be explained by the increased risk of infection faced by frontline workers due to repeated exposure, work in high-risk environments, and frequent interaction with infected patients. Moreover, the inherently hazardous nature of medical work, especially in the context of airborne and respiratory diseases like COVID-19, further elevates the risk of severe outcomes among this group.

In this study, the hospital of admission was identified as a significant predictor of ICU admission and mortality among COVID-19 patients. Specifically, Dura Hospital recorded the highest percentage of ICU admissions and deaths, whereas Red Crescent Hospital had the lowest. This disparity may be explained by several factors. Dura Hospital, located in the southern region, served a broader catchment area and admitted a higher volume of critically ill patients during the study period. In contrast, Red Crescent Hospital was newly established, had limited ICU capacity, admitted predominantly non-critical cases, and operated with a smaller and less experienced medical staff. These contextual differences in infrastructure, case severity, and clinical capacity likely contributed to the observed variations in outcomes across hospitals (19).

The results of the current study demonstrated a statistically significant association between blood group and ICU admission among COVID-19 patients. This finding aligns with the results of (20), who also reported higher ICU admission rates among patients with blood group A. A possible explanation is that individuals with blood group O, considered universal donors, tend to exhibit more favorable immune responses and overall physiological resilience compared to those with blood group A. Blood groups are determined by the presence of specific antigens on the surface of red blood cells (RBCs), which may influence susceptibility to certain infections, including SARS-CoV-2. The A blood group, in particular, may be associated with increased vulnerability to severe outcomes, including ICU admission. However, it is important to note that some studies have not found a statistically significant relationship between blood group and the risk of ICU admission, such as the findings reported by (21).

In this study, obesity was found to be significantly associated with ICU admission among COVID-19 patients, aligning with findings reported by Kim et al. (18). Obesity is already recognized as a major risk factor for severe COVID-19 outcomes due to its frequent association with comorbid conditions such as cardiovascular disease, diabetes, and hypertension. Furthermore, obese individuals often face physiological complications that can exacerbate disease severity, including obstructive sleep apnea—which may increase pulmonary hypertension—and a higher body mass index (BMI), which can complicate clinical management in hospital settings. These challenges may be further compounded by chronic low-grade inflammation and immune dysregulation commonly seen in obese patients. However, it is worth noting that not all studies have reported a statistically significant relationship between obesity and ICU admission; for example, Plataki et al. (22) did not find such an association in their analysis (22).

Our findings revealed a statistically significant relationship between smoking status and ICU admission among COVID-19 patients, corroborating the study by Kim et al. (18). Smoking is a well-established risk factor that contributes to increased severity of pulmonary infections through mechanisms such as airway damage, lung inflammation, and diminished respiratory and immune function. Moreover, smoking is linked to a range of comorbidities, including cardiovascular disease, chronic obstructive pulmonary disease (COPD), lung cancer, and diabetes, all of which may compound the risk of critical illness in COVID-19 patients (23). Smoking has also been associated with more severe influenza infections requiring hospitalization, which may similarly affect COVID-19 outcomes. One proposed mechanism involves smoking-induced overexpression of angiotensin-converting enzyme 2 (ACE2) receptors in the lungs, which are known to facilitate SARS-CoV-2 cellular entry and viral propagation, thereby increasing the likelihood of ICU admission and mortality (18).

Kim et al. (18) reported a statistically significant association between pregnancy and ICU admission among COVID-19 patients, which aligns with the findings of the current study. Pregnancy induces various physiological and immunological changes that may increase susceptibility to infectious diseases, including COVID-19. Pregnant women may experience more severe manifestations of respiratory illnesses due to altered immune responses and cardiopulmonary adaptations. Additionally, pregnancy-related complications such as gestational diabetes and hypertension may further exacerbate the severity of COVID-19, increasing the risk of ICU admission and potentially mortality. These overlapping risk factors underscore the need for heightened surveillance and targeted care strategies for pregnant women infected with SARS-CoV-2.

Some studies have also discussed that pregnant women experience a significant increase in blood volume, particularly plasma, by up to 50% around the 34th week of gestation. This physiological change ensures adequate perfusion of the uterus, fetus, and placenta. However, the resultant fluid overload can lead to edema—a condition that may compromise respiratory function and increase vulnerability to infections, including COVID-19. Such changes may heighten the likelihood of ICU admission among pregnant women (18). Moreover, according to Lokken et al. (24), pregnant individuals with COVID-19 are at higher risk for severe outcomes, including hospitalization and intensive care, particularly in the third trimester, which coincides with peak hemodynamic changes (24).

Although numerous previous studies have reported a statistically significant association between advanced age and ICU admission among COVID-19 patients (2, 24–26), the current study did not observe a significant relationship between age and ICU admission. This may be explained by the relatively homogenous age distribution in our sample, with most patients falling within the middle-age range (35–65 years), limiting the statistical contrast between younger and older groups. Additionally, ICU admission criteria were based primarily on clinical deterioration rather than chronological age, as outlined in the Ministry of Health protocols, which may have attenuated age-related effects observed in other settings.

In this study, the majority of participants were female (*n* = 120, 60%). However, gender was not found to be statistically associated with ICU admission among COVID-19 patients. This finding contrasts with the results of Kaeuffer et al. (25) and Noor and Islam (26), who reported male gender as a significant risk factor for severe COVID-19 outcomes and ICU admission. The absence of a significant association in our study may be due to the relatively balanced distribution of male and female participants across ICU and non-ICU groups, which could diminish any observable differences. Consequently, gender alone may not serve as a reliable predictor of ICU admission in this particular cohort of hospitalized COVID-19 patients.

The results showed no statistically significant association between educational level and ICU admission among COVID-19 patients (*p* = 0.112). In this study, 69 participants had higher education, 8 were illiterate, 63 had primary education, and 60 held a diploma or bachelor’s degree. Although participants with higher education represented a notable proportion (34.5%), this did not translate into significant differences in ICU admission rates. This finding aligns with broader literature suggesting that while education is often associated with general health outcomes, its direct effect on ICU admission during COVID-19 may be mediated by other factors such as comorbidities and healthcare access (27–29).

This study found no statistically significant association between social status and ICU admission among COVID-19 patients. However, previous research has shown that the quality of life during periods of widespread social restrictions can be influenced by factors such as education level, employment status, and financial stability. In particular, the pandemic exacerbated existing burdens on married women due to their multifaceted roles as caregivers, homemakers, and often the primary managers of household responsibilities. With the imposition of lockdowns and the closure of schools and public facilities, married women were disproportionately affected by increased domestic workload and childcare demands. These added responsibilities have been linked to deteriorating mental health, manifesting as heightened stress, anxiety, depression, and emotional fatigue (30).

The results of this study showed no statistically significant association between place of residence and ICU admission among Palestinian COVID-19 patients. Similarly, monthly income did not demonstrate a significant relationship with ICU admission. This may be attributed to the fact that all patients were admitted to governmental hospitals, where the treatment protocol is standardized and uniform regardless of socioeconomic status. Consequently, disparities in healthcare access and treatment quality based on income were minimized. However, it is worth noting that patients from higher-income brackets may have had better access to certain medications not readily available in government hospitals, such as Tocilizumab (Actemra), which could influence outcomes in subtle ways. Recent literature supports the notion that socioeconomic inequalities may influence outcomes in broader healthcare settings, but such effects may be attenuated in standardized public health systems (31).

This study found no significant relationship between patients’ medical history (i.e., comorbidities) and ICU admission or death among COVID-19 patients in the West Bank, Palestine. This contrasts with findings from other international studies, such as those conducted in France, the United States, and Bangladesh, which reported a strong link between comorbidities and severe COVID-19 outcomes (18, 25, 26). One possible explanation for this discrepancy is the age distribution of our sample. The majority of ICU-admitted patients in this cohort were relatively young, ranging from 35 to 45 years old. The uniformity of age distribution across groups may have minimized the expected effect of comorbidities. Additionally, critical illness in these patients may have been driven more by aggressive immune responses, such as cytokine storm syndrome, rather than underlying chronic diseases (32). It is also notable that other laboratory markers—such as elevated CRP, ferritin levels >900 μg/L, D-dimer >1,500 μg/L, body temperature >39°C, respiratory rate >30, and oxygen saturation <88%—were frequently abnormal but could not be included in the analysis due to incomplete data. Nonetheless, these markers were commonly elevated in available records and might be more reflective of ICU risk in this younger population. Recent studies have also begun to highlight that even previously healthy individuals can experience severe disease due to dysregulated immune responses, especially in regions with limited access to early diagnostics and interventions (33).

The current study revealed no statistically significant association between COVID-19 patients’ chief complaints and their rates of ICU admission or in-hospital mortality, which aligns with the findings of Weehuizen and Hoepelman (47). However, this lack of association should be interpreted with caution. It may be attributable to the relatively limited sample size, which restricts the statistical power needed to detect associations—particularly for symptoms with lower prevalence. Additionally, retrospective data collection may have led to underreporting or inconsistent documentation of presenting complaints, despite our efforts to supplement missing data through manual review.

Nonetheless, certain physiological indicators showed significant differences. Oxygen saturation (O₂ saturation, *p* = 0.040) and partial pressure of oxygen (PaO₂, *p* = 0.010) were both significantly lower among ICU-admitted patients, supporting their value as early markers of clinical deterioration. These findings are consistent with those of Sadeghi et al. (34), who emphasized the prognostic value of declining oxygen levels. Notably, “silent hypoxia”—characterized by severe hypoxemia without overt respiratory distress—has been documented in up to 30% of hospitalized COVID-19 cases and is strongly associated with poor outcomes (35).

Furthermore, blood urea nitrogen (BUN) levels were significantly associated with ICU admission (*p* = 0.017), supporting the findings of Küçükceran et al. (35). BUN reflects both renal function and catabolic stress, with elevated levels often seen in conditions like heart failure, infection-related hypercatabolism, and gastrointestinal bleeding (34). Similarly, white blood cell count (WBC) showed significant association (*p* = 0.032), consistent with observations by Sadeghi et al. (34). In COVID-19, leukocyte dysregulation—particularly the failure of monocytes and macrophages to contain SARS-CoV-2 replication—has been implicated in disease progression. The random blood sugar (RBS) levels also showed significant association with ICU admission (*p* = 0.002), echoing the results of Lotfy and Shama (36), who emphasized that severe COVID-19 can cause stress-induced hyperglycemia and pancreatic dysfunction (36).

Furthermore, blood urea nitrogen (BUN) levels were significantly associated with ICU admission (*p* = 0.017), supporting the findings of Küçükceran et al. (35). BUN reflects both renal function and catabolic stress, with elevated levels often seen in conditions such as heart failure, infection-related hypercatabolism, and gastrointestinal bleeding (34). Similarly, white blood cell count (WBC) showed a significant association (*p* = 0.032), consistent with observations by Sadeghi et al. (34), as leukocyte dysregulation—particularly the failure of monocytes and macrophages to contain SARS-CoV-2 replication—has been implicated in disease progression. Random blood sugar (RBS) levels also showed a significant association with ICU admission (*p* = 0.002), echoing the results of Lotfy and Shama (36), who emphasized that severe COVID-19 can trigger stress-induced hyperglycemia and pancreatic dysfunction.

Although elevated RBS was significantly associated with ICU admission, the strength and direction of this association varied across subgroups and analytic models. This may reflect the influence of factors such as steroid-induced hyperglycemia, unmeasured baseline glycemic control, or variability in timing of glucose measurement relative to disease progression. These complexities underscore the need for future studies that incorporate baseline glycemic status and treatment effects when evaluating the prognostic value of blood glucose in COVID-19 patients.

Importantly, both RBS and certain medications such as Actemra (tocilizumab) represent modifiable risk factors, and their associations with ICU admission warrant further exploration within a causal inference framework. Recent methodological advances, including target trial emulation (TTE), provide a structured approach to estimating causal effects from observational data by defining eligibility criteria, treatment strategies, follow-up windows, and outcome measures, mimicking a randomized controlled trial (37). Applying such methods—e.g., marginal structural models or inverse probability weighting—can help determine whether interventions targeting glycemic control or immunomodulation reduce ICU admissions. These tools are particularly relevant in real-world and resource-limited settings like Palestine, where randomized trials may be logistically or ethically challenging.

The presence of pneumonia was another key predictor, as patients with pneumonia were significantly more likely to be admitted to the ICU (*p* = 0.003), in line with findings from Sadeghi et al. (34) and Küçükceran et al. (35). COVID-19-related pneumonia contributes to acute respiratory distress syndrome (ARDS) and cardiovascular complications, which often necessitate critical care (48). In contrast, variables such as systolic/diastolic blood pressure, blood pH, and bicarbonate (HCO₃) levels were not significantly different between ICU and ward patients, contradicting studies like Ran et al. (38), which highlighted their prognostic relevance (34, 35, 38).

Antibiotic use presented a mixed picture. While vancomycin and meropenem usage were associated with higher ICU admission rates, Actemra (tocilizumab) appeared to have a protective effect, although only 13 patients received it. This result aligns with Minihan et al. (39), who noted that tocilizumab is beneficial when combined with corticosteroids in patients requiring oxygen support (39). However, the small sample size limits the strength of this conclusion. Antibiotics such as ceftriaxone and azithromycin did not show a significant association with ICU admission, consistent with Abu-Rub et al. (40), who noted that antibiotics are ineffective against viral infections unless complicated by bacterial coinfections (40).

Regarding corticosteroids, although dexamethasone was widely used and is known to reduce mortality in patients requiring respiratory support, the present study’s findings differ from those of Reyes et al. (41), who reported it as a risk factor for ICU admission (41). Obesity emerged as a significant predictor of ICU admission, which is in agreement with Singh et al. (42). The infiltration of SARS-CoV-2 into adipocytes likely contributes to an exaggerated inflammatory response and poorer outcomes in obese individuals (18). Conversely, Singh et al. (49) did not find obesity to be a significant predictor, highlighting variability across populations (42, 43).

The WBC and RBS levels were also confirmed as independent predictors of ICU admission (*p* = 0.005 and *p* = 0.014, respectively), supporting Sobhani et al. (50). These markers may reflect heightened immune and metabolic stress in severely ill patients. Notably, this study also observed an inverse association between higher BUN/RBS levels and ICU admission, a finding inconsistent with studies by Cerbu et al. (44), who identified them as strong predictors. These discrepancies may be due to patient heterogeneity or methodological differences (44).

Although vomiting was not statistically significant (*p* = 0.40), the adjusted odds ratio (OR = 1.508) suggested a possible trend toward worse outcomes. Moreover, obese patients exhibited significantly shorter ward stays prior to ICU transfer, consistent with Kim et al. (18), suggesting that obesity accelerates disease progression. On the contrary, Singh et al. (49) did not find obesity to impact ICU timing.

PaO₂ and BUN levels were also linked to earlier ICU admission, reinforcing the findings of Küçükceran et al. (35) and Sadeghi et al. (34). Uremic toxicity and oxygen desaturation act as early warning signs of decompensation (42). Similarly, RBS levels were associated with earlier ICU transfer, as glucose dysregulation, especially indiabetics, is known to exacerbate COVID-19 severity (36, 50).

Patients with pneumonia experienced earlier ICU admissions than those without pneumonia, consistent with Russo et al. (45). COVID-19-induced lung injury can precipitate rapid clinical decline. Pregnant women also had significantly shorter times to ICU transfer, in line with Kim et al. (18), likely due to physiological changes such as increased cardiac output and reduced pulmonary reserve (45).

Smokers were also admitted to ICU sooner than non-smokers, reflecting existing literature by Guan et al. (2) and Kim et al. (18). Smoking is linked to immune suppression and increased ACE2 expression, which facilitates viral entry. However, this contradicts findings from Maraqa et al. (51), who reported no significant association. Lastly, individuals with blood type A+ reached the ICU more quickly than those with O+, corroborating Halim et al. (20), who proposed that blood group antigens may influence coagulation and inflammatory pathways.

This study represents the first comprehensive investigation in Palestine specifically designed to identify the clinical, demographic, and treatment-related risk factors associated with ICU admission among hospitalized COVID-19 patients. It provides localized evidence tailored to the Palestinian healthcare context, which is crucial for guiding national health policy and resource allocation. By employing both logistic and Cox regression analyses, this study adds to the global understanding of COVID-19 severity predictors, particularly in low-resource and conflict-affected settings. The findings—especially those related to obesity, blood urea nitrogen (BUN), white blood cell (WBC) count, and random blood sugar (RBS)—can be immediately utilized by healthcare professionals to triage and prioritize care for high-risk patients.

However, the study also faced several limitations. Incomplete laboratory test data for some patients arose due to limited hospital capabilities; many analyses were performed externally and results were communicated informally (e.g., via WhatsApp). Missing or partial CT scan reports reduced the accuracy of radiological assessments. The exclusion of children and adolescents limits the generalizability of findings across all age groups. Furthermore, since the study focused solely on hospitalized adults, there is a risk of selection bias in estimating illness severity. Some patient files lacked key information such as clinical complaints, medical history, and vital signs, which reduced the robustness of certain statistical comparisons. Incomplete laboratory test data for some patients arose due to limited hospital capabilities, as many advanced analyses (e.g., CRP, ferritin, D-dimer, IL-6) were either unavailable or outsourced with results communicated informally. This missingness may not be random and could disproportionately affect patients from under-resourced hospitals, introducing potential selection bias. The absence of such critical biomarkers may have limited our ability to identify or confirm additional predictors of ICU admission. Furthermore, it may have attenuated the strength or statistical significance of certain associations. Future studies should ensure routine collection and centralized availability of key laboratory parameters to strengthen the predictive validity of ICU risk models.

The number of patients who received Tocilizumab (Actemra) was small (n = 13), which limited the study’s ability to evaluate its potential protective effects. In addition, political constraints—particularly Israeli-imposed delays and obstructions in the delivery of essential medical supplies, including ventilators—compromised the continuity of care and follow-up in critically ill patients. Time limitations also necessitated reliance on archived paper records for over two-thirds of the sample, except in Hugo Chavez Hospital where electronic data were available. Finally, the study cohort consisted entirely of moderate to critical cases, which limited variance in outcome comparisons.

Another important consideration is the inherent heterogeneity of COVID-19 patient populations. Our cohort included individuals with varied sociodemographic characteristics, comorbidities, and treatment exposures across six hospitals with differing capacities. Such diversity introduces potential effect modification and may explain inconsistencies with findings from other settings. Prior literature has emphasized that risk factors for ICU admission and outcomes can vary across subgroups defined by age, sex, comorbidity burden, or even ethnicity (46). Future studies using stratified analyses or interaction models—and ideally grounded in frameworks like subgroup-specific target trial emulation—are warranted to explore how clinical predictors vary across different patient populations. These approaches will enhance the precision and applicability of ICU triage strategies in diverse healthcare contexts.

Additionally, this retrospective study is subject to potential selection bias, as only hospitalized patients with complete records were included, possibly excluding individuals with milder disease managed in outpatient settings. Moreover, confounding by indication may have influenced observed associations between certain treatments (e.g., corticosteroids, immunomodulators) and ICU admission, since such interventions were often initiated based on clinical severity. While we adjusted for key confounding variables in multivariable models, residual or unmeasured confounding cannot be entirely ruled out.

Future research should include pediatric and adolescent populations to expand generalizability. Nationwide implementation of standardized electronic medical records is essential to reduce missing data and enhance the quality of retrospective analyses. Multicenter prospective studies are needed to validate the predictive models identified in this study. Routine availability of key laboratory markers (e.g., BUN, CRP, ferritin, d-dimer) and imaging data should be ensured in all treatment centers. Broader access to treatments such as Tocilizumab should be secured and studied in larger cohorts. International and local stakeholders must collaborate to remove barriers to the timely provision of essential medical equipment in Palestine. Finally, policymakers can use the findings from this study to develop early-warning systems or clinical scoring tools for ICU triage in resource-constrained settings.

In conclusion, the current study identifies key clinical, demographic, and biochemical predictors of ICU admission among hospitalized COVID-19 patients in the West Bank, Palestine. Obesity, abnormal WBC, BUN, and RBS levels, as well as pneumonia and low PaO₂, were significantly associated with both increased risk and earlier need for intensive care. These findings offer critical, context-specific insights that can guide evidence-based triage protocols, optimize resource allocation, and inform national health strategies in Palestine and similar low-resource settings.

Furthermore, our findings could establish a data base for new similar future pandemics and/or resurge of COVID-19 for better emergency preparedness and intervention measures mainly due to the risk of emergence of new variants and the decline in the surveillance system and wane herd immunity in the population which could re-emerge COVID-19 outbreaks.

## Data Availability

The raw data supporting the conclusions of this article will be made available by the authors, without undue reservation.

## Glossary

ACE2: Angiotensin-Converting Enzyme 2
ALT: Alanine Aminotransferase
AST: Aspartate Aminotransferase
AOR: Adjusted Odds Ratio
BUN: Blood Urea Nitrogen
CI: Confidence Interval
COVID-19: Coronavirus Disease 2019
CRP: C-Reactive Protein
CT: Computed Tomography
HR: Hazard Ratio
ICU: Intensive Care Unit
INR: International Normalized Ratio
IRB: Institutional Review Board
LDH: Lactate Dehydrogenase
MOH: Ministry of Health
OR: Odds Ratio
PCR: Polymerase Chain Reaction
PaO₂: Partial Pressure of Arterial Oxygen
PaCO₂: Partial Pressure of Carbon Dioxide
PT: Prothrombin Time
PTT: Partial Thromboplastin Time
RBS: Random Blood Sugar
RBC: Red Blood Cell
SARS-CoV-2: Severe Acute Respiratory Syndrome Coronavirus 2
SpO₂: Peripheral Oxygen Saturation
WBC: White Blood Cell
